# Urinary lumirubin excretion in jaundiced preterm neonates during phototherapy with blue light-emitting diode vs. green fluorescent lamp

**DOI:** 10.1038/s41598-023-45147-7

**Published:** 2023-10-26

**Authors:** Yumiko Uchida, Yukihiro Takahashi, Chikara Kurata, Yukihiro Morimoto, Eishin Ohtani, Asako Tosaki, Akiko Kumagai, Peter Greimel, Toshiya Nishikubo, Atsushi Miyawaki

**Affiliations:** 1 Division of Neonatal Intensive Care, Maternal, Fetal and Neonatal Medical Center, Nara Medical University Hospital, 840 Shijo-Cho, Kashihara-City, Nara 634-8521 Japan; 2 Central Clinical Laboratory, Nara Medical University Hospital, Kashihara-City, Nara Japan; 3 R&D Division, Ushio Inc, Himeji-City, Hyogo Japan; 4 SANKEN, Osaka University, Ibaraki-City, Osaka Japan; 5 Laboratory for Cell Function Dynamics, RIKEN Center for Brain Science, Wako-City, Saitama Japan; 6 Biotechnological Optics Research Team, RIKEN Center for Advanced Photonics, Wako-City, Saitama Japan

**Keywords:** Biomarkers, Diseases

## Abstract

Phototherapy converts lipophilic unconjugated bilirubin to hydrophilic bilirubin photoisomers, such as lumirubin. We comparatively used a blue light-emitting diode (LED) and a green fluorescent lamp (FL) as light sources for phototherapy of hyperbilirubinemic preterm neonates with the aim of examining potential differences in urinary lumirubin excretion between these two wavelengths. Urinary lumirubin levels were measured using a fluorescence assay with blue light exposure in the presence of the unconjugated bilirubin-inducible fluorescent protein UnaG, and denoted as urinary UnaG-bound bilirubin (UUB)/creatinine (Cr) (μg/mg Cr). Preterm neonates born at ≤ 33 weeks gestational age and treated with phototherapy were subjected to this study. The maximum UUB/Cr level during phototherapy per device intensity was compared between neonates treated with the blue LED and the green FL. A total of 61 neonates were examined to determine the maximum UUB/Cr levels. The median of maximum UUB/Cr excretion per light intensity of each device (μg/mg Cr/μW/cm^2^/nm) was 0.83 for the blue LED and 1.29 for the green FL (*p* = 0.01). Green light was found to be more effective than blue one for bilirubin excretion via urinary lumirubin excretion. This is the first spectroscopic study to compare the efficacy of phototherapy at different wavelengths using fluorescence assay.

## Introduction

In 2022, the American Academy of Pediatrics revised its clinical practice guideline, stating that intensive phototherapy (PT) requires a narrow-spectrum light-emitting diode (LED) that produces blue-range light at a central wavelength of approximately 475 nm with an irradiance of at least 30 µW/cm^2^/nm^1^. In Japan, green-range (490–550 nm) light as well as blue-range (430–490 nm) light have commonly been in clinical use, on the basis of a finding that the green spectrum is more conducive to the production of lumirubin, which is also called EZ-cyclobilirubin, the primary bilirubin photoisomer^2^. While bilirubin photoisomers have only been measured using high-performance liquid chromatography (HPLC) to date, we recently developed a simple bedside method that quantitatively measures lumirubin levels by using UnaG, which becomes fluorescent upon binding to unconjugated bilirubin specifically^3,4^. This method is now dubbed PUZZLU (Photo-isomerization in the presence of UnaG to ZZ-bilirubin for Lumirubin). In the present study, we applied PUZZLU to a large number of urine samples from patients to statistically demonstrate that lumirubin excretion is more efficient by green-light PT than blue-light PT.

## Results

Sixty-one patients ultimately participated in the study. Thirty-two patients received PT with the blue LED and 29 with the green FL. Neonatal parameters are shown in Table 1. There were no significant differences in gestational age and birth weight between the two groups (Mann–Whitney *U* test).

**Table 1:** Characteristics of hyperbilirubinemic neonates included in the study.

	Blue LED (n = 32)	Green FL (n = 29)	*p* value
Sex (male/female) (number)	20/12	19/10	–
Gestational age (weeks)	31.4 ± 0.4 (26.5–33.4)	30.6 ± 0.4 (26.0–33.4)	0.83
Birth weight (g)	1423 ± 77 (650–2224)	1354 ± 83 (500–1976)	0.42
Time after initiation of PT (h; postnatal)	33.8 ± 3.4 (18.0–94.2)	30.9 ± 5.0 (9.0–129.5)	0.80
Time of PT application (h; postnatal)	26.4 ± 2.2 (18.0–62.1)	39.5 ± 4.6 (10.0–95.0)	0.13
PT duration required for UUB/Cr to reach its highest value (h)	13.4 ± 1.6 (2.5–40.5)	13.5 ± 3.0 (3.0–62.5)	0.31

Continuous data are presented as the median ± standard error and range in parentheses. Data were analyzed using the Mann–Whitney *U* test. *p* values < 0.05 were considered statistically significant.

*ED* light-emitting diode, *FL* fluorescent lamp, *PT* phototherapy, *UUB* urinary UnaG-bound bilirubin, *Cr* creatinine.

### Light intensities of PT devices

**Figure 1:**
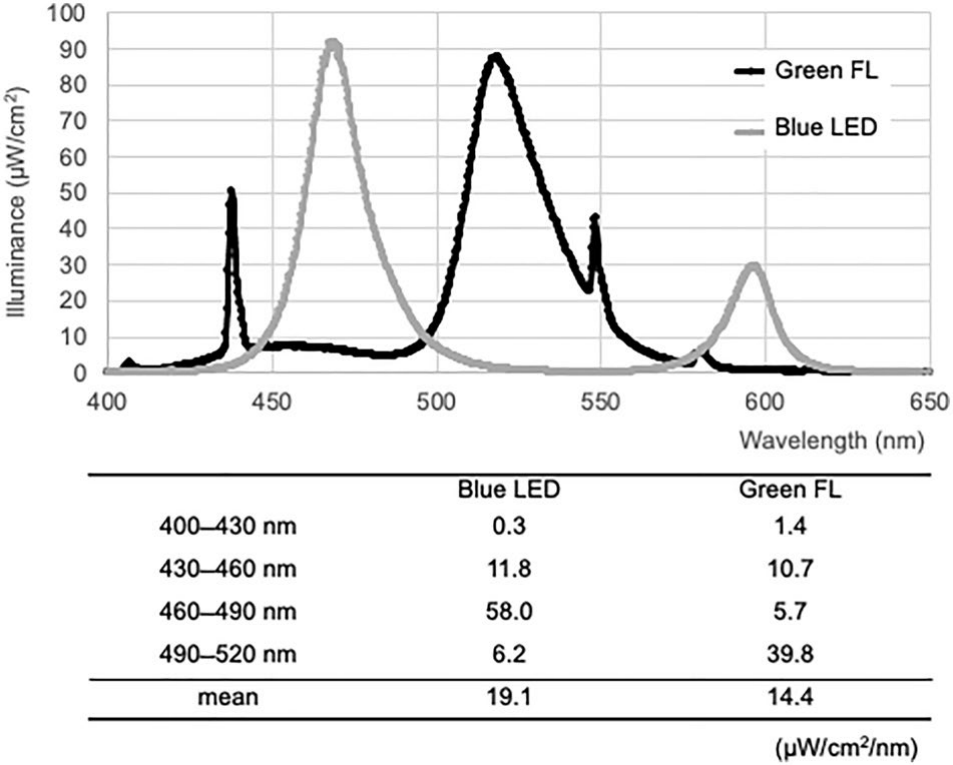
Average light intensity per wavelength band. The average light intensity between 430 and 460 nm, within which the maximum absorption wavelength of ZZ-bilirubin is contained, was almost the same for blue LED and green FL. In contrast, the average light intensity was higher between 460 and 490 nm for blue LED and between 490 and 520 nm for green FL.

The wavelength range (with peak emissions) of blue LED was 410 ~ 632 nm (470 nm), and that of green FL was 400 ~ 600 nm (518 nm) (shown in Fig. 1). The average light intensities of the blue LED at bandwidths of 400–430 nm, 430–460 nm, 460–490 nm, and 490–520 nm were 0.3, 11.8, 58.0, and 6.2 µW/cm^2^/nm, respectively. Similarly, the average light intensities of the green FL at the same respective bandwidths were 1.4, 10.7, 5.7, and 39.8 µW/cm^2^/nm. In particular, the average light intensity between 400 ~ 520 nm through the skin^5^ was 19.1 and 14.4 µW/cm^2^/nm for the blue LED and green FL, respectively. And the photon quantities were 5.4 × 10^15^ and 4.3 × 10^15^ photons/cm^2^/s for the blue LED and green FL, respectively.

### UUB excretion and TSB decline

For the blue LED and green FL groups, the median initiation times of PT (postnatal) were 33.8 and 30.9 h, respectively. Likewise, the PT duration times were 26.4 and 39.5 h, and the PT times taken for UUB/Cr to reach its highest value were 13.4 and 13.5 h, respectively. None of these parameters showed significant differences between the two groups. The therapeutic effects of the device per average light intensity were compared between the blue LED and green FL groups. The medians (ranges) of TSB reduction rate divided by the average light intensity (%/h/μW/cm^2^/nm) were 49.2 (− 24.9–173.0) and 47.2 (− 97.9–198.4), respectively, with no significant difference (*p* = 0.54, Mann–Whitney *U* test).

### Maximum UUB excretion per average light intensity between 400 ~ 520 nm

**Figure 2:**
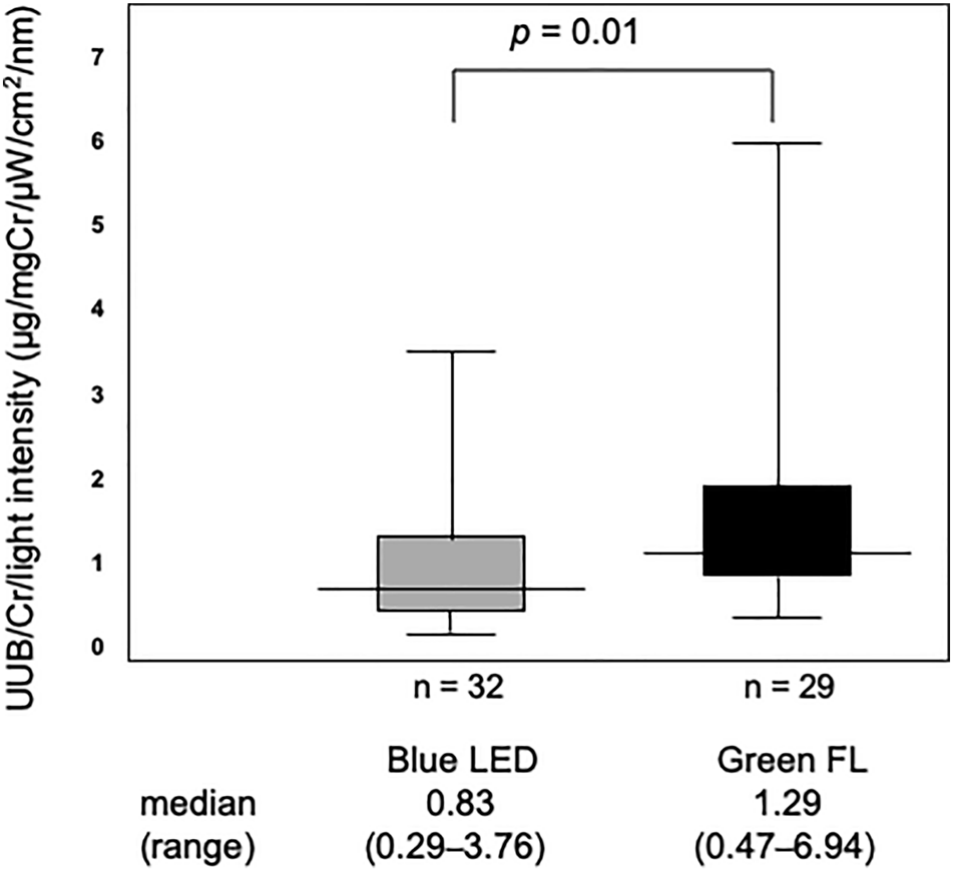
Maximum UUB excretion per average light intensity between 400 and 520 nm. The maximum UUB levels per average light intensity between 400 and 520 nm transmitted through neonatal skin were compared between blue LED and green FL. There was difference between the two and more UUB was excreted with green FL.

The medians (ranges) of maximum UUB/Cr excretion per light intensity of the device (μg/mg Cr/μW/cm^2^/nm) were 0.83 (0.29–3.76) for the blue LED group and 1.29 (0.47–6.94) for the green FL group. The green FL group showed significantly higher maximum UUB/Cr levels per light intensity from 400 to 520 nm of the PT device (*p* = 0.01, Mann–Whitney *U* test) (shown in Fig. 2).

## Discussion/conclusion

The formation of lumirubin and its excretion into urine (although it is said to account for only one-fifth of bile excretion^2^) are thought to contribute to the elimination of bilirubin from infants receiving PT^2,6^. Although lumirubin in specimens is usually measured by HPLC, this measurement requires expensive equipment and special expertise. We previously harnessed a ZZ-bilirubin-inducible fluorescent protein, UnaG, to develop a fluorometric method, called PUZZLU (Photo-isomerization with UnaG to ZZ-bilirubin for detecting LUmirubin)^4^. In this method, specimens are irradiated by blue light in the presence of UnaG, and lumirubin is photo-isomerized back to ZZ-bilirubin, which immediately renders UnaG green fluorescent through high-affinity binding^4^. We compared PUZZLU with liquid chromatography–mass spectrometry (LC–MS/MS) and found that lumirubin measured by PUZZLU amounted to approximately 40% of that measured by LC–MS/MS^4^. Since the amount ratio was very reproducible, we believe that PUZZLU is an easy and effective method for bedside monitoring of urinary lumirubin. In the present study, we practically used PUZZLU to comparatively evaluate the effectiveness of blue vs. green wavelengths of PT.

While there is a worldwide guideline for PT that treats neonatal jaundice, the optimum conditions of photoirradiation have remained undetermined^5^. An in vitro study by Vreman et al. showed that bilirubin degradation and lumirubin production occurred most efficiently by light of 490–500 nm^7^. However, Ebbessen et al. found no significant differences in serum concentrations of total bilirubin isomers or ZZ-bilirubin in patients irradiated at 497 nm vs. 459 nm^8^. Although Ennever et al.^9^ examined the wavelength dependency of the conversion of bilirubin to EZ-bilirubin and then EZ-cyclobilirubin (lumirubin), it is still uncertain how deep blue and green lights penetrate tissue for effective photoconversion in situ.

Although the green FL contains only a small fraction (10%) of the light component between 460 and 490 nm, which is currently considered to be effective for PT, it yielded higher excretion efficiency than the blue LED in the present study. Importantly, while approximately 70% of the light from the green FL consists of a long-wavelength spectrum with a peak at 518 nm, it also contains a significant peak at 436 nm that is characteristic of mercury. It is noted that this wavelength approximates the maximum absorption wavelength of bilirubin. We thus suspect this component substantially contributes to the great excretion of lumirubin by the green FL.

Bilirubin (ZZ-bilirubin) was long considered only a non-functional waste substance associated with liver disease or even a potentially neurotoxic substance^10^. However, recent studies^11,12^ have shown that ZZ-bilirubin may protect us from disease as one of the most intrinsic antioxidants in the body. And lumirubin, a more hydrophilic bilirubin photoisomer generated by PT, is regarded to be less toxic compared to ZZ-bilirubin, yet retains its antioxidant capacity. Nevertheless, another study^13^ found that lumirubin had a significant effect on the expression of major pro-inflammatory genes in different cell models of central nervous system origin. Thus, similar to ZZ-bilirubin, it is possible that lumirubin behaves as a yin-yang molecule with both beneficial and harmful effects.

In recent years, potentially harmful effects of PT in small premature neonates have been reported^14–19^. Therefore, the physiological effects of lumirubin need to be explored more extensively with easier monitoring than before. Our simple method, PUZZLU, for lumirubin measurement in urine is expected to promote large-scale follow-up studies of PT-treated infants with hyperbilirubinemia.

A limitation of this study is that it was designed in an actual neonatal care practice, i.e., it is not a comparative study using light sources with precisely designed wavelengths and light intensity. A more rigorous control of wavelength range would be needed to study the effect of color on lumirubin production.

## Materials and methods

### Subjects

Preterm neonates were enrolled at the neonatal intensive care unit at Nara Medical University Hospital, Japan, between October 2018 and May 2021. Selected were ≤ 33 weeks of gestational age and had uncomplicated hyperbilirubinemia. Neonates eligible for this study were subjected to single-color PT; they were randomly divided into two groups for either blue LED exposure (neoBlue in High Mode, Natus Medical, San Carlos, CA) or green fluorescent lamp (FL) exposure (FL20S-BG-NU (20 W × 5), PIT-220TLR, Atom Medical Corp., Tokyo, Japan). Of the 106 neonates, 38 showed resistant hyperbilirubinemia after single PT and had to be switched to dual PT (simultaneous exposure to both the blue LED and green FL). Seven neonates did not provide both pre-PT and post-PT urine samples. As a result, a total of 61 neonates were included in this study. Decisions regarding the initiation and termination of PT were made based on the standard clinical criteria^20^.

### Phototherapy devices and light intensities

Spectral intensities were determined by integrating each spectrum as a function of wavelength measured using an HR4000 spectrometer (Ocean Optics, Dunedin, FL) calibrated with Excel spreadsheet software (Microsoft Excel for Mac, ver. 16.68, Microsoft Co., Redmond, WA). Light intensity was measured at a distance of 30 cm in both devices. As light is most transmissive in the range of 400–520 nm in the epidermis^5^, the light intensity was examined over four sub-ranges; 400–430, 430–460, 460–490, and 490–520 nm (shown in Fig. 1). The spectral intensities of the wavelength bands were integrated (μW/cm^2^) in an Excel spreadsheet and divided by 30 nm to obtain the average light intensity. Also, we measured the illuminated spectra by using the HR spectrometer (Ocean Optics), in which the light energy of each one nano-meter (µW/cm^2^/nm) are plotted as a function of wavelength of incident light. The energy is related to photon number as the following equation,

$$\mathrm{E}=n\times h \frac{c}{\lambda },$$

where E is the energy in Joule, *n* is the photon quantities, *h* is Planck's constant 6.626 $\times$ 10^−34^ $J\cdot s$, *c* is the speed of light (2.998 $\times$ 10^17^ nm/s), and *λ* the wavelength of light in nm. Therefore, the photon quantities of each 1 nm in spectra measured can be calculated, and the photon quantities of desired spectrum region are obtained by summation between 420 and 520 nm.

### Blood samples

Blood samples were collected from patients by pricking the heel into sodium heparinized micro-hematocrit capillary tubes (Becton, Dickinson and Company, Franklin Lakes, NJ). Serum was immediately separated by centrifugation for 5 min at 11,800 × *g* (KUBOTA3220, Kubota Corp., Tokyo, Japan) for measurement of total serum bilirubin (TSB) using 2-wavelength (455 nm, 575 nm) spectrophotometry (BL-300, TOITU Co., Tokyo, Japan). Blood collection was executed within 2 h of the initiation of PT for measurement of pre-PT TSB or > 2 h after PT initiation for mid-PT TSB.

### Urine samples

All procedures related to the preparation of samples and bilirubin were performed in a dimly lit room in the basement.

Urine samples were collected by placing several small cotton balls near the external genitalia in a diaper. After removal, the cotton balls were immediately squeezed to transfer the urine into a micro-tube (ST-0150R, INA, OPTICA Co., Ltd, Osaka, Japan), and the sample was frozen at − 80 °C until use. Urine samples were centrifuged for 10 min at 2600 × *g* (KUBOTA 2800, Kubota Corp., Tokyo, Japan) at 4 °C, and the supernatants were subjected to PUZZLU assay. Urinary creatinine was measured using an enzymatic assay kit (sarcosine oxidase–peroxidase) (Serotec Co., Ltd., Sapporo, Japan). Urine collection was performed three times everyday from postnatal day 0 (P0) to P7.

### Bilirubin (ZZ-BR) solution

Reference bilirubin (ZZ-BR) solutions were prepared to generate a standard curve for the measurement of urinary UnaG-binding bilirubin (UUB). A total of 2 mg of bilirubin (98%) (FUJIFILM Wako Pure Chemical Co., Osaka, Japan) was dissolved in 2 mL of 0.1 mol/L NaOH, which was immediately neutralized with 1 mL of 0.1 mol/L phosphoric acid and then mixed with 7 mL of human serum albumin (HSA; albumin 5% I.V. 5 g/100 mL, Japan Blood Products Organization, Tokyo, Japan). The concentration of bilirubin in the preparation was measured using a BL-300 2-wavelength spectrophotometer (TOITU Co., Ltd., Tokyo, Japan) (455 nm, 575 nm). The bilirubin solution was diluted with phosphate-buffered saline (0.1 mol/L, pH 7.2) to desired concentrations. The above reagents, except HSA, were purchased from FUJIFILM Wako Pure Chemical Co. (Osaka, Japan).

### UnaG mixture

UnaG is a fluorescent protein isolated from Japanese eel muscle^3^. Its apo-form, apoUnaG, was prepared as a purified bacterially expressed recombinant protein. The “UnaG mixture” contained apoUnaG, HSA, and ascorbic acid at a ratio of 1:1:1 (v/v/v), in which their final concentrations were 5 µmol/L, 0.01%, and 0.1%, respectively^4^. L ( +)-ascorbic acid was purchased from FUJIFILM Wako Pure Chemical Co. and added to reduce photo-oxidation of bilirubin and its derivatives.

### PUZZLU

**Figure 3:**
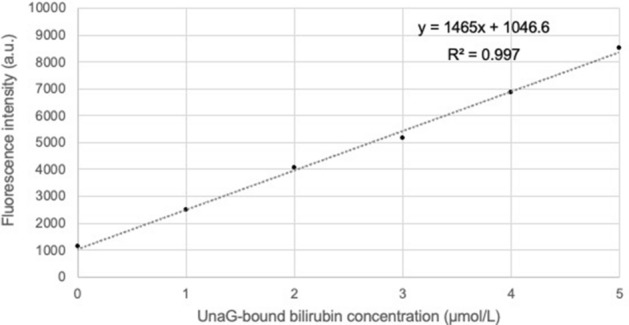
Calibration curve for UnaG-bound bilirubin. Urinary lumirubin is reconverted to ZZ-bilirubin by exposure to blue light in the presence of UnaG and detected as UnaG-bound bilirubin. Therefore, a calibration curve for UnaG-bound bilirubin using a bilirubin reagent (ZZ-bilirubin) must be prepared in advance.

A black microplate (Microtest™ 96-well assay plate, black, flat bottom, BD Biosciences, Franklin Lakes, NJ) was prepared, and 50 µL of each sample (reference bilirubin or urine) was added to 150 µL of UnaG mixture. The resulting 200-µL reaction mixture was pipetted into a well of the microplate. The fluorescence intensity of each well was measured spectrophotometrically at 37 °C with fluorescence filters for excitation and emission at 498 and 527 nm, respectively. For calibration curve generation, the bilirubin solution was diluted with PBS to concentrations of 0, 1, 2, 3, 4, and 5 µmol/L and analyzed as described above. Before light exposure, the calibration curve was drawn as UnaG-bound bilirubin (shown in Fig. 3).

Thawed urine samples were exposed to blue-light LED irradiation (P4630-blue LED unit; wavelength range 420–520 nm, with a peak emission at 450 nm, 10.5 mW/cm^2^ at a distance of 4 cm above the microplate, Ushio Inc., Tokyo, Japan). Samples were assayed in duplicate every 15 min (until 90 min) or 30 min (over 90 min). The UUB concentration was defined as the highest fluorescence intensity during the exposure time, as determined from the calibration curve. Urine sampled before PT was undiluted, whereas that during PT was diluted fivefold. Final UUB levels were corrected for urinary creatinine and expressed as UUB/Cr (µg/mg Cr).

### Calculations

The rate of TSB reduction was expressed as 1 − (post-TSB/pre-TSB) × 100/ (PT exposure time) (%/h). Light in the range of 400 to 520 nm entering the epidermis is mostly transmitted^5^. Therefore, the TSB reduction rate was divided by the average light intensity from 400 to 520 nm to express the therapeutic efficiency of the PT device (%/h/μW/cm^2^/nm). Similarly, maximum UUB/Cr was also divided by the average light intensity from 400–520 nm to express the excretion efficiency of the phototherapy device (μg/mg Cr/μW/cm^2^/nm).

### Statistical analysis

Linear regression analyses and Mann–Whitney *U* tests were performed using StatFlex software, ver. 6 (Artech Co., Osaka, Japan).

### Ethics declarations

The study protocol was reviewed and approved by the Nara Medical University ethics committee (approval no. 1033) and performed in accordance with relevant guidelines. Written informed consent was obtained from the patents of each neonate included in the study.

## Data Availability

All data generated or analyzed during this study are included in this published article.
